# Analysis of hypoxia-inducible factor alpha polyploidization reveals adaptation to Tibetan plateau in the evolution of schizothoracine fish

**DOI:** 10.1186/s12862-014-0192-1

**Published:** 2014-08-28

**Authors:** Lihong Guan, Wei Chi, Wuhan Xiao, Liangbiao Chen, Shunping He

**Affiliations:** Institute of Hydrobiology, Chinese Academy of Sciences, Wuhan, Hubei P. R. China; University of Chinese Academy of Sciences, Beijing, P. R. China; College of Fisheries, Huazhong Agricultural University, Wuhan, Hubei P. R. China; College of Fisheries and Life Science, Shanghai Ocean University, Shanghai, P. R. China

**Keywords:** HIF-α, Schizothoracine fish, Hypoxia, Positive selection, Tibetan Plateau adaptation

## Abstract

**Background:**

Hypoxia-inducible factor (HIF) is a master regulator that mediates major changes in gene expression under hypoxic conditions. Though HIF family has been identified in many organisms, little is known about this family in schizothoracine fish.

**Results:**

Duplicated *hif-α* (*hif-1αA*, *hif-1αB*, *hif-2αA*, and *hif-2αB*) genes were identified in schizothoracine fish. All the deduced HIF-α proteins contain the main domains (bHLH-PAS, ODDD, and TAD), also found in humans. Evidence suggests a Cyprinidae-specific deletion, specifically, a conserved proline hydroxylation motif LxxLAP, in the NODD domain of schizothoracine fish HIF-1αA. In addition, a schizothoracine-specific mutation was observed in the CODD domain of the specialized and highly specialized schizothoracine fish HIF-1αB, which is the proline hydroxylation motif mutated into PxxLAP. Standard and stochastic *branch-site* codon model analysis indicated that only *HIF-1αB* has undergone positive selection, which may have led to changes in function. To confirm this hypothesis, HIF-αs tagged with Myc were transfected into HEK 293 T cells. Each HIF-1αB was found to significantly upregulate luciferase activity under normoxic and hypoxic conditions, which indicated that the HIF-1αB protein was more stable than other HIF-αs.

**Conclusions:**

All deduced HIF-α proteins of schizothoracine fish contain important domains, like their mammalian counterparts, and each HIF-α is shorter than that of human. Our experiments reveal that teleost-specific duplicated *hif-α* genes played different roles under hypoxic conditions, and HIF-1αB may be the most important regulator in the adaptation of schizothoracine fish to the environment of the Tibetan Plateau.

**Electronic supplementary material:**

The online version of this article (doi:10.1186/s12862-014-0192-1) contains supplementary material, which is available to authorized users.

## Background

Gene duplication was first proposed by Bridges in 1936 in a study of the fruit fly *Drosophila melanogaster* [1]. It was further detailed by Ohno in a book, *Evolution by Gene Duplication*, in 1970 [2]. The ancestor of vertebrates experienced two rounds of whole-genome duplication which may have played a key role in adaptive evolution of vertebrate [2–5]. The third round of whole-genome duplication that occurred in teleosts is thought to have driven the diversification of teleosts [6–9].

Oxygen is critical to all aerobic life, particularly for animals living in water, which contains only 1/30th the oxygen of air at the same partial pressure [10–13]. Sophisticated cellular mechanisms have evolved to allow organisms to detect and react to changes in oxygen levels. In these mechanisms, oxygen acts as the terminal electron acceptor in the mitochondrion of metazoans [11,14,15]. When studying the expression of EPO from Hep3B cells under hypoxic conditions, Semenza and Wang [16] identified a nuclear factor bound to the hypoxia-inducible enhancer (HIE) of the erythropoietin (EPO) gene at a site required for hypoxia activation of transcription. This nuclear factor was named hypoxia-inducible factor (HIF). In mammals, HIF mediates major changes in gene expression under hypoxic conditions and participates in a series of processes including angiogenesis, erythropoiesis, glucose and iron transport, glycolysis, and cell-cycle control [11,17–21]. HIF is a heterodimeric transcription factor of the bHLH-PAS (basic helix-loop-helix-per-ARNT-sim) family. It consists of an erratic alpha subunit (such as HIF-1α) and a steady beta subunit (such as HIF-1β or ARNT) [11,19,22–26]. According to studies in *Caenorhabditis elegans* and *D. melanogaster*, there is only one HIF-α in invertebrates, but in vertebrates, there are at least three functional HIF-α isoforms: HIF-1α, which regulates the acute hypoxic response; HIF-2α, which regulates the chronic hypoxic response; and HIF-3α, which inhibits the activities of the other two isoforms [11,14,19,20,27,28]. HIF-1α and HIF-2α both have two oxygen-dependent degradation domains (NODDD and CODDD) that are located in the central region, and two transactivation domains—an inner activation domain (N-TAD) that overlaps with the CODDD and a carboxy-terminal activation domain (C-TAD). HIF-3α lost the C-TAD [14,19,22,29]. With adequate oxygen (normoxia), one or both of the highly conserved prolyl residues located in ODDD of HIF-α (HIF-1α and HIF-2α) becomes hydroxylated by the prolyl-4-hydroxylase (PHD), and then the hydroxylated HIF-α interacts with the von-Hippel-Lindau tumor suppressor (VHL) and recruits ubiquitin ligase, which is consequently degraded by the proteasome [19,22,30,31]. However, under hypoxic conditions, HIF-α, which is stabilized, translocates to the nucleus *via* its nuclear localization signal (NLS) motif, where it dimerizes with HIF-β into HIF heterodimer and binds to the core DNA motif (G/ACGTG) in hypoxia-response elements (HREs) for transcriptional activation of the target genes [19,22,32,33].

**Figure 1:**
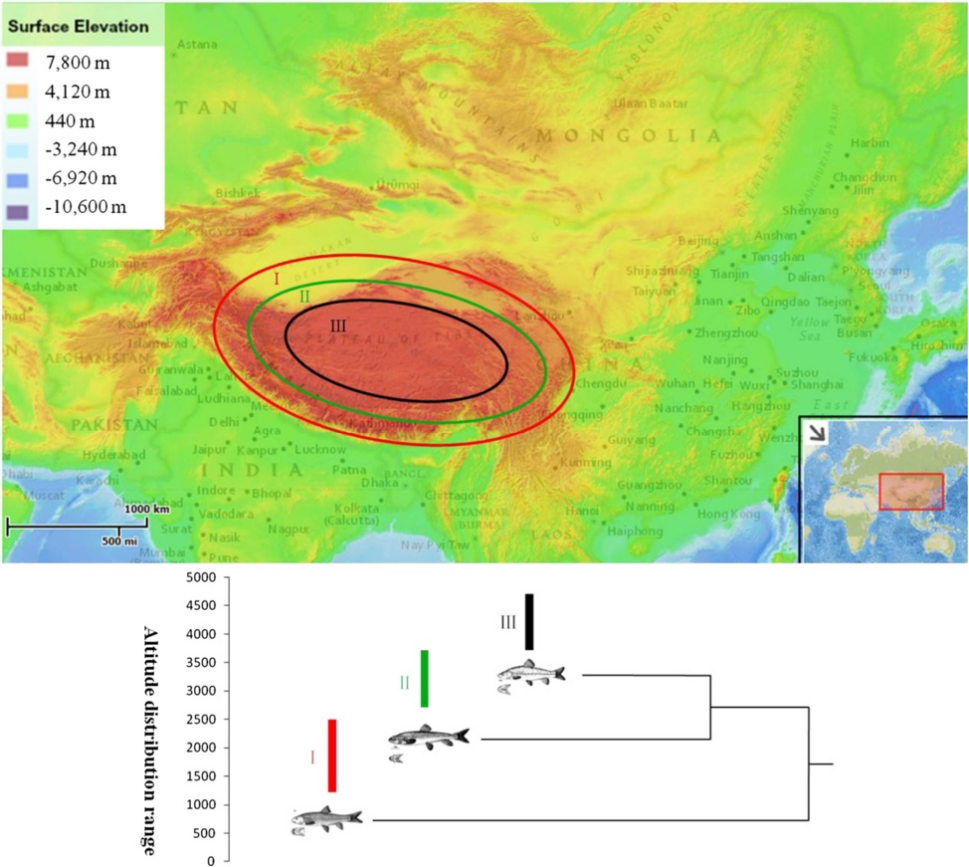
**Altitude distribution of three groups of schizothoracine fish in Tibetan Plateau.** The map was downloaded from the National Geographic ArcMap Esri db on June 22, 2014 [79]. The solid boxes indicate the altitude distribution range of three groups of the main Schizothoracine genera. I: primitive schizothoracine fish, II: specialized schizothoracine fish, III: highly specialized schizothoracine fish.

The teleost-specific whole-genome duplication that occurred early in the teleost evolution generated six *hif-α* genes: *1A/B*, *2A/B*, and *3A/B*, which was followed by the loss of one member of each *A/B* paralogous pair in most euteleosts but not in cyprinids [20]. The schizothoracine fish (Teleostei: Cyprinidae), which are divided into three groups (primitive, specialized, and highly specialized) according to the differences in their scales, pharyngeal teeth, and barbels, are the endemic and most diverse group of cyprinids distributed in the Tibetan Plateau and its adjacent areas. The elevation in this area ranges from 700 to 5000 m, and the environment is characterized by hypoxia and low temperatures [34–36]. Primitive schizothoracine fish including *Schizothorax*, *Schizocypris* (not found in China), and *Aspiorhynchus*, are distributed at the edge of the Tibetan Plateau. Specialized schizothoracine fish included *Diptychus*, *Ptychobarbus*, and *Gymnodiptychus*, and highly specialized schizothoracine fish included *Gymnocypris*, *Oxygymnocypris*, *Schizopygopsis*, *Platypharodon*, *Chuanchia*, and *Herzensteinia*. The distribution of specialized and highly specialized schizothoracine fish is largely limited to the central region of the plateau [37]. Each group represents specific periods of geological evolution of the Tibetan Plateau, which ultimately caused the modern distribution pattern of schizothoracine fish, in which primitive, specialized, and highly specialized schizothoracine fish are distribued from the edges to the central part of the plateau (Figure 1) [35]. Previous analyses of the mitochondrial genome have demonstrated that schizothoracine fish, which are teleost fishes, are well-adapted to high altitudes and hypoxia [38]. However, *hif-α* genes, which are related to hypoxia, have been little studied in schizothoracine fish. Chi [12] referred to the evolutionary patterns of the *hif-α* genes in two high-altitude fish, but no signs of adaptive evolution was detected since the species limitation.

To better understand the relationship between adaptation to hypoxia and *hif-α* genes in plateau and plains fish, teleost-specific *hif-α* gene duplications (*hif-1αA*/*B* and *hif-2αA*/*B*) were cloned and identified in six species of schizothoracinae and in zebrafish. Further computational and experimental analyses reveal the mode of evolution and response to hypoxia of these gene paralogs.

## Results

### Characterization of *hif-α* duplications and phylogenetic analysis

Although many studies have focused on HIF in cyprinids, duplicated HIF-α paralogs have never been identified in schizothoracine fish. The sequences of *hif-α* paralogs (*hif-1αA/B* and *hif-2αA/B*) of the six species of schizothoracinae were obtained here for the first time [GenBank: KJ679876-KJ679899]. The N-terminal of zebrafish *hif-1αA* sequence was also isolated [GenBank: KJ679875]. The data sets supporting the results of this article are also available in the Dryad Digital repository [39]. The lengths of different *hif-α* paralogs in schizothoracine fish were found to vary. However, the lengths of specific *hif-α* genes remained consistent among highly specialized schizothoracine fish, even when the schizothoracine fish came from different genera (Additional file 1: Table S1).

**Figure 2:**
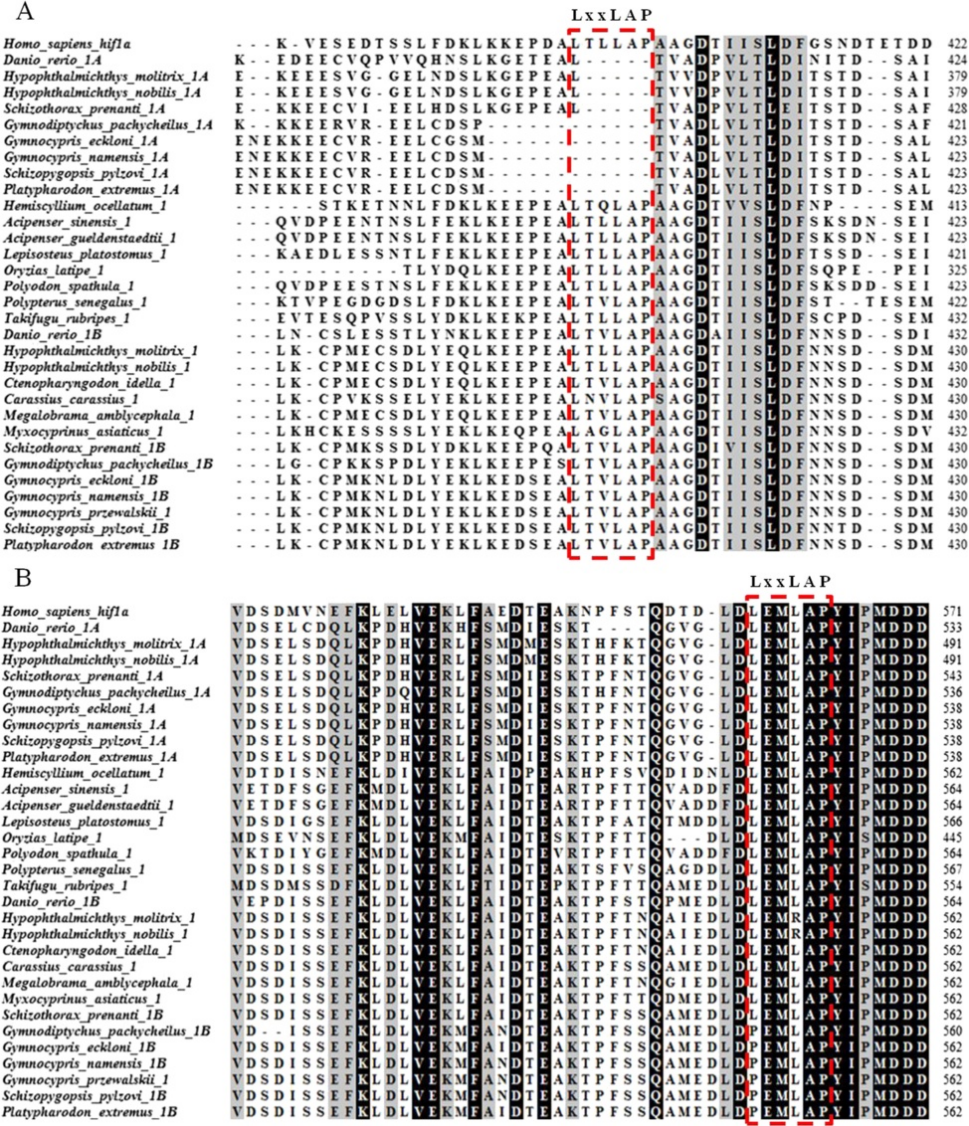
**Partial multiple sequence alignment of the deduced HIF-1α protein sequences. A)** In Cyprinidae, HIF-1αA proteins were found to have a deletion (LxxLAP) in the conserved NODD domain. **B)** Specialized and highly specialized schizothoracine fish were found to have a mutation (LxxLAP to PxxLAP) in the conserved CODD domain of HIF-1αB. Dashes indicate that the gaps inserted for facilitated alignment. The two conserved proline hydroxylation motif LxxLAP are indicated by the dotted boxes.

Protein analysis showed that HIF-α paralogs from schizothoracine fish all contained the main domains, like their mammalian counterparts. The similarity between duplicated HIF-1αA and HIF-1αB was 52%, and the similarity between duplicated HIF-2αA and HIF-2αB was 55%. The bHLH-PAS domain of HIF-αs N-terminal was highly conserved, but the C-terminal was less conserved, especially near the oxygen-dependent proline hydroxylation sites. A deletion, specifically, the conserved proline hydroxylation motif LxxLAP, was detected in the NODD domain of HIF-1αA in all six schizothoracine fish and zebrafish (Figure 2 and Additional file 2: Figure S1 A). Currently, *hif-1αA* was only amplified in Cyprinidae, so this deletion might be Cyprinidae-specific. Sequence alignment suggested that there was a schizothoracine-specific mutation in the CODD domain of the HIF-1αB of specialized and highly specialized schizothoracine fish, specifically the proline hydroxylation motif mutated into PxxLAP (Figure 2 and Additional file 2: Figure S1 B). All deletions and mutations were located in functional ODD domains that interacted with PHD via the PHD-HIF oxygen-sensing system [11,22,40]. This may affect function during biological processes. The targets of PHD in mammals are proline 402 and proline 564 in the ODD domain, but Pro-564 is the primary critical substrate in PHD binding to HIF-1α [10,41]. It is here speculated that HIF-1αB is more likely to change function than other HIF-αs are.

**Figure 3:**
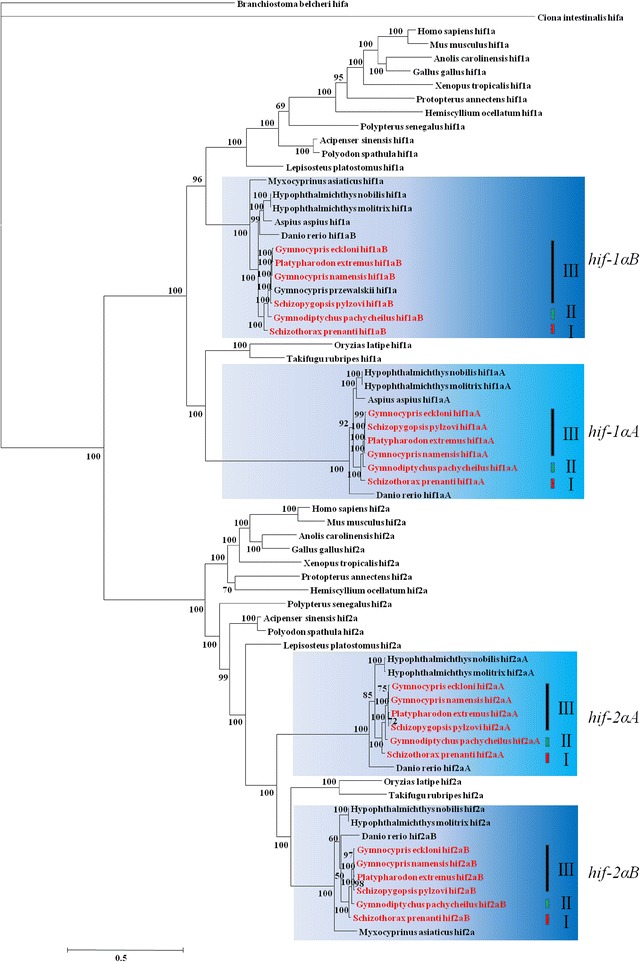
**Phylogenetic tree was constructed for the selected chordate** ***hif-1α*** **and** ***hif-2α*** **genes.** The phylogenetic tree was constructed using MrBayes with GTR + I (0.0821) + G (1.0813) modes. To search tree space, Markov chain Monte Carlo (MCMC) simulations were performed for 2,000,000 generations with parameter sampling every 1,000 generations. The first 1,000 samples were discarded during the burn-in phase of the MCMC analysis. The Bayesian posterior probability values are indicated beside the branches.

The chordate phylogenetic tree of *hif-1α* and *hif-2α* genes was reconstructed using the MrBayes and RAxML softwares with sea squirt and amphioxus *hif-α* sequences as the outgroups [42,43]. The two computer programs inferred similar tree topologies. The Bayesian posterior probability values and maximum likelihood bootstrap values were high (Figure 3, Additional file 3: Figure S2). The topology was largely consistent with that found in previous studies [11,29]. However, the phylogeny estimated for the *hif-1α* gene was inconsistent with the recently published *hif-1α* phylogenies based on the PhyloBayes [44] and RAxML softwares [20]. Recent results indicated a sister relationship between *hif-1αA* and *hif-1αB*, our results indicate instead that *hif-1αB* might be more similar to the ancient *hif-1α* than *hif-1αA* is. Massey *et al.* [45] and Roje [46] have researched on the effects of non-neutral gene on phylogeny. In this study, more *hif-1αB* sequences were used than any other studies, which might have led to a non-synonymous to synonymous rate ratio (dN/dS) distinct from that obtained in previous studies. The phylogeny (Additional file 4: Figure S3 A) in the schizothoracine fish constructed using concatenated *hif-1α* and *hif-2α* sequences agrees with that using mitochondrial cytochrome b [38,47]. For this reason, it was considered suitable for use in the following selection pressures analysis.

### Molecular evolution of the teleostei HIF-α duplicate

First, standard *branch-site* models in PAML [48] were used to assess the different selective pressures experienced by the protein coding sequences of each *HIF-α* paralogous pair. In this model, the *foreground* branches are the branches tested for positive selection, and the *background* branches are all other branches on the tree [48]. In the likelihood ratio test (LRT), *branch-site* model A is the alternative model allowing ω_2_ to vary among codons, while the simpler null model is model A but with ω_2_ = 1 fixed [48]. In this study, three methods were used to specify the *foreground* branches. According to the LRT of *branch-site* models, for *HIF-1αB*, when the clade of all schizothoracine fish was specified as the *foreground* branches, the likelihood obtained under model A was not significantly higher than that obtained under the null hypothesis of no positive selection; but when the clade of specialized and highly specialized schizothoracine fish was specified as the *foreground* branches, the likelihood obtained under model A was significantly higher than that of the null model (2Δln*L* = 12.16, *p* < 0.01, *df* = 1) and ω > 1 (ω = 7.11, Table 1). When only the clade of highly specialized schizothoracine fish was specified as the *foreground* branches, the likelihood obtained under model A was quite significantly higher than that of the null model (2Δln*L* = 19.79, *p* < 0.01, *df* = 1) and ω > 1 (ω = 23.24) (Table 1). The posterior probability of sites that had undergone positive selection was calculated using the Bayes empirical Bayes (BEB) method in model A. For these last two analyses, there were 9 and 10 amino acid sites in *HIF-1αB* sequences with a posterior probability greater than 0.5. In the second method, the amino acid sites at position 464 (according to *HIF-1αB* of *D. rerio*) had a posterior probability 0.990 as indicated by BEB, which was significant at the 1% level, but in the third method, the amino acid sites at position 387 and 510 had a posterior probability 0.989 and 0.987 as indicated by BEB, which was significant at the 5% level. In this way, they were identified as crucial amino acid sites that had experienced positive selection. Three of the 9 sites and 4 of the 10 sites were located in the ODD domain by the last two methods. For *HIF-1αA*, *HIF-2αA*, and *HIF-2αB*, no other positively selected site was detected.

**Table 1:** **Positively selected sites detected in** ***HIF-1αB*** **of specialized and highly specialized schizothoracine fish by** ***branch-site*** **models**

**Foreground branch**	**Δln** ***L***	**Parameter estimates**	**Positive sites**
specialized and highly specialized schizothoracine fish	6.08	*p*_0_ = 0.820 p_1_ = 0.149 p_2_ = 0.026 *ω*_0_ = 0.074 *ω*_1_ = 1 *ω*_2_ = 7.113	215P 370E 371 T 386P 387E 396S 464D^**^ 510D 534I
highly specialized schizothoracine fish	9.89	*p*_0_ = 0.820 p_1_ = 0.17 p_2_ = 0.010 *ω*_0_ = 0.079 *ω*_1_ = 1 *ω*_2_ = 23.246	41H 370E 371 T 386P 387E^*^ 428S 464D 467P 510D^*^ 648 T

Note: * mean pp > 95%, ** mean pp > 99%.

Secondly, stochastic *branch-site* models in fitmodel [49] were used to detect the different selective pressures experienced on the protein coding sequences of *HIF-1α* and *HIF-2α*, respectively. For *HIF-1α*, the likelihood obtained under M3 + S1 (ω2 = 8.7857) model was significantly higher than that obtained under null (M1 + S1) model with ω2 > 1 and 2ΔlnL = 6.29 (p < 0.05). However, there were only 3 amino acid sites (77 K, 395S, and 398 L, according to *HIF-1αB* of *D. rerio*) with a posterior probability > 0.5. For *HIF-2α*, no other positively selected site was detected.

In standard *branch-site* models, although the *foreground* branches specified for *HIF-1αB* were different, the last two methods showed similar positively selected sites with different levels of significance. With stochastic *branch-site* models, positive selection was also detected in *HIF-1αA* and *HIF-1αB*. It is here hypothesized that the *HIF-1αB* experienced strong selective pressure, which may have led to changes in function. The higher the altitude at which the schizothoracine fish were distributed, the more significant the selective pressure was. This may be one of the mechanisms by which schizothoracine fish have adapted to the high altitudes of the Tibetan Plateau.

### Expression and hypoxic regulation of different HIF-α paralogs in HEK 293 T cells

**Figure 4:**
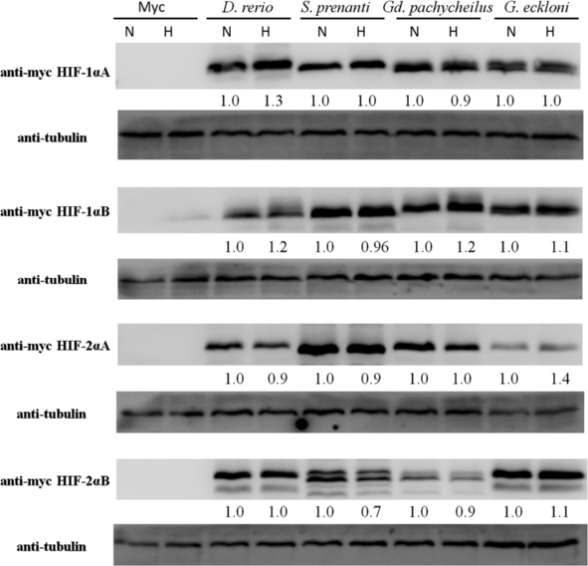
**Expression of HIF-α in HEK 293 T cells under normoxic and hypoxic conditions.** N: normoxia, H: hypoxia. There was no significant different in protein expression levels under hypoxic and normoxic conditons, excepting that there was more expression of HIF-1αA and HIF-1αB of *D. rerio*, HIF-1αB of *Gd. Pachycheilus* and HIF-2αA of *G. eckloni* under hypoxic conditions.

RytKönen [20] showed that the transcription of *hif-1α* decreased after hypoxic insult in adult zebrafish, but the transcription of *hif-2αA* remained stable and *hif-2αB* transcription increased significantly. To determine whether changes in oxygen tension regulate HIF-α at the protein level, HEK 293 T cells were transfected with either the indicated Myc-tagged HIF-α constructs or an empty vector, and cells were subjected to 18 h of hypoxic treatment after 6 h of normoxic cultivation. Western blot analysis indicated that all the fish HIF-αs could be expressed in HEK 293 T cells. Compared to the normoxic condition, there were only rarely significant increases in levels of protein expression under hypoxic conditions: HIF-1αA and HIF-1αB in *D. rerio*, HIF-1αB in *Gd. pachycheilus* and HIF-2αA of *G. eckloni* (Figure 4). Generally, the expression level of HIF-1α was greater than that of HIF-2α after hypoxic treatment and under normoxic conditions (Additional file 5: Figure S4).

**Figure 5:**
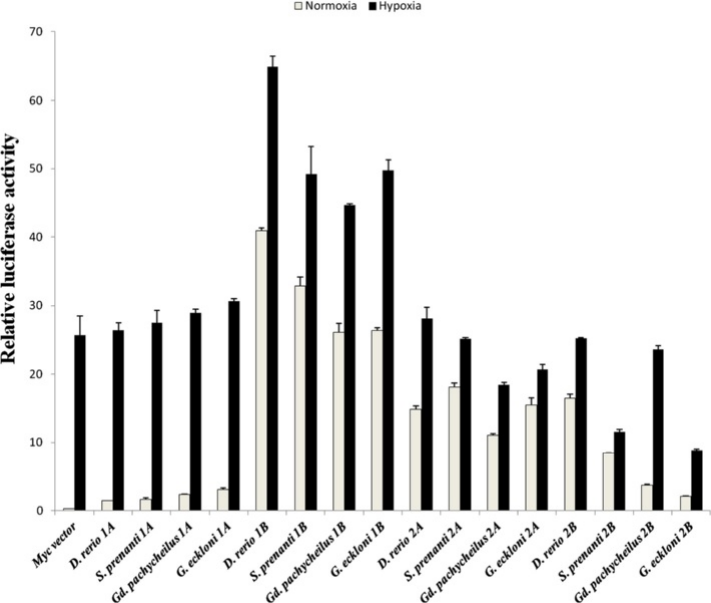
**Transcriptional activity of HIF-αs under normoxic and hypoxic conditions.** Each HIF-1α and HIF-2α was found to upregulate luciferase activity under normoxia, and the luciferase activity of HIF-1αB was high even under normoxia.

To determine whether selective pressure could have affected the transcriptional activity of HIF-α toward their common targets, empty vector (control) and the indicated Myc-tagged HIF-α constructs were co-transfected with the HRE-luciferase reporter and pTK-*Renilla* luciferase in HEK 293 T cells. Each HIF-1α and HIF-2α was found to upregulate luciferase activity under normoxic conditions (Figure 5). This indicated that the function of fish HIF-αs had been conserved. In addition, HIF-1αB showed more luciferase activity than other HIF-αs, even under normoxic conditions, suggesting that HIF-1αB protein may be more stable. The transcriptional activity of HIF-2α was obvious under normoxic conditions but not detectable under hypoxic conditions.

## Discussion

Fish possess homologs of HIF-α and HIF-β that share strong similarity with those of humans. They may play roles similar to those of their mammalian counterparts in expression of oxgen-dependent genes [13]. The sequence of *hif-α* gene had been extensively characterized in fishes. The first fish *hif-α* gene was identified in rainbow trout (*Oncorhynchus mykiss*) in 2001 [50]. The sequence was slightly shorter than that of mammals. It included bHLH-PAS and ODD domains and proline and asparagine residues, which were relatively conserved in the deduced rainbow trout HIF-α protein. Subsequently, *hif-α* gene was reported from killfish (*Fundulus heteroclitus*), grass carp (*Ctenopharyngodon idellus*), crucian carp (*Carassius carassius*), zebrafish (*Danio rerio*), Atlantic croaker (*Micropogonias undulatus*), asp (*Aspius aspius*), three-spined stickleback (*Gasterosteus aculeatus*), Russian sturgeon (*Acipencer gueldenstaedtii*), naked carp (*Gymnocypris przewalskii*), Wuchang bream (*Megalobrama amblycephala*), Chinese sucker (*Myxocyprinus asiaticus*), bighead carp (*Hypophthalmichthys nobilis*), silver carp (*Hypophthalmichthys molitrix*), Prenant’s schizothoracin (*Schizothorax prenanti*), Namucuo naked carp (*Gymnocypris namensis*), and so on [10,12,51–58].

Although previous studies identified HIF-α in schizothoracine fish, the present study is the first to identify teleost-specific duplicated HIF-α paralogs. It not only provides new information for HIF research in teleosts but also supplies a foundation for further study of the adaptation of schizothoracine fish to high altitudes and low oxygen levels in the Tibetan Plateau. Each schizothoracine fish HIF-α was smaller than the corresponding human HIF-α. The HIF-1αA/B and HIF-2αA/B of schizothoracine fish shared more than 46 and 53% similarity with human HIF-1α and HIF-2α, respectively. HIF-1αB and HIF-2αB shared more sequence identities with human HIF-1α and HIF-2α than HIF-1αA and HIF-2αA did in all species except *Gd. pachycheilus*. The bHLH-PAS domain of HIF-αs N-terminal was highly conserved, but the ODD domain of the C-terminal was less conserved, especially in regions in the vicinity of the oxygen-dependent proline hydroxylation sites. A Cyprinidae-specific deletion (LxxLAP) in the NODD domain of HIF-1αA may have occurred in all six schizothoracine fish, and a schizothoracine-specific mutation LxxLAP mutated to PxxLAP in the CODD domain of the specialized and highly specialized schizothoracine fish HIF-1αB. The HIF hydroxylase pathway was regulated by the PHD family of oxygen-dependent prolyl hydroxylases in metazoans [19]. In mammals, the Pro-564 in the CODD domain is the primary critical substrate in PHD binding to HIF-1α [31]. It was here deduced that although the deletion and mutation were all located in the ODD domain, the change in the CODD domain of HIF-1αB was more important than the deletion in the NODD domain of HIF-1αA was in the evolution of schizothoracine fish.

The HIF pathway plays a pivotal role in the response to hypoxia, and the PHD family of oxygen-dependent prolyl hydroxylases plays a critical role in regulating HIF stability [19]. Some candidate genes associated with the HIF pathway showed signals of positive selection in species living in the Tibetan Plateau. For instance, *EPAS1* (*HIF-2α*), *EGLN1*, and *PPARA* were detected in Tibetans [59,60]; *ADAM17* in the yak [61]; *ADORA2A*, *CCL2*, *ENG*, *PIK3C2A*, *PKLR*, *ATP12A*, and *NOS3* in the Tibetan antelope [62]; *SRF*, *TXNRD2*, and *WNT7B* in the ground tit [63]; and *EPAS1*, *SIRT7*, *PLXNA4*, and *MAFG* in the Tibetan mastiff [64]. In this study, computational estimation suggested that the specialized and highly specialized schizothoracine fish *HIF-1αB* have experienced significantly selective pressure. Positively selected sites were detected in *HIF-1α*, *HIF-2α*, and *HIF-2α*, respectively in schizothoracine fish, Tibetans, and Tibetan mastiff. This shows that fish and mammals developed different mechanisms for adapting to the special environment of the Tibetan Plateau.

Functional analysis was performed by transfecting schizothoracine fish HIF-α in HEK 293 T cells. Results showed that the expression level of HIF-1α differed from that of HIF-2α. Hypoxia was found to increase the abundance of HIF-1αA/B but not that of HIF-2αA/B. The decrease in the protein levels of HIF-2αA/B may be attributable to the combined impact of the reduced protein synthesis under hypoxic conditions and the rapid folding rate of HIF-α protein in mammalian cells [65]. The luciferase assay showed that all the HIF-αs characterized in this study were capable of forming functional heterodimers with human HIF-β and could activate HRE reporter gene, but they performed different levels of oxygen-dependent regulation. Each HIF-α (HIF-1αA/B and HIF-2αA/B) was able to upregulate luciferase activity under normoxic conditions, and HIF-1αB was more significant, suggesting that HIF-1αB was more stable under normoxic conditions. The transcriptional activity of HIF-1αB was higher than that of other HIF-1αs under hypoxic conditions, and transcriptional activity was stronger under hypoxic conditions than under normoxic conditions. These results were also supported by structural analysis. For HIF-1αB, the bHLH-PAS domain, which is responsible for heterodimerization with HIF-β and DNA binding, was conserved; and the ODD domain, which interacts with PHD, was mutated [10,19,22,66,67]. It was also discovered that HIF-2αA/B downregulated luciferase activity under hypoxic conditions. This was consistent with the decrease observed in protein levels of HIF-2αA/B after hypoxia treatment.

## Conclusions

The teleost-specific duplicated *hif-α* paralogs, *hif-1αA*, *hif-1αB*, *hif-2αA*, and *hif-2αB*, were successfully identified in schizothoracine fish. Each deduced HIF-α was found to have the same principal domains as its mammalian counterparts, and had evolved specialized roles in the response to hypoxia. It is here postulated that HIF-1αB may be the most important regulator in the adaptation of schizothoracine fish to the environment of the Tibetan Plateau. Although sequence variation and significant selective pressure were detected in HIF-1αB of the specialized and highly specialized schizothoracine fish, transcriptional activity showed no significant difference between plateau fish (schizothoracine fish) and plains fish (zebrafish). This might be because zebrafish are also hypoxia tolerant fish [10,13]. Further studies are needed to explore the structure and functions of schizothoracine fish HIF-αs and to determine their roles in the mechanism underlying the response to hypoxic conditions. The adaptation to Tibetan Plateau of schizothoracine fish involves a complicated evolutionary mechanism, which is affected by a cluster of genes. In the future, research on transcriptome and genome of schizothoracine fish may contribute to explain the mechanism of their adaption to Tibetan Plateau.

## Methods

### Ethics statement

The experiments were performed in accordance with the Ethics Committee of the Institute of Hydrobiology, Chinese Academy of Sciences. The policies were enacted according to Chinese Association for Laboratory Animal Sciences, and coordinated with the Institutional Animal Care and Use Committee (IACUC) protocols [68,69].

### Sampling

*Schizothorax prenanti* (primitive schizothoracine fish) and *Gymnodiptychus pachycheilus* (specialized schizothoracine fish) were collected from Ya’an in Sichuan Province; *Gymnocypris eckloni*, *Schizopygopsis pylzovi*, and *Platypharodon extremus* (highly specialized schizothoracine fish) were collected from the headwaters of the Yellow River; *Gymnocypris namensis* (highly specialized schizothoracine fish) was collected from Namtso Lake; *Danio rerio* (AB strain) was collected from Institute of Hydrobiology, Chinese Academy of Sciences. Permissions for extracting the fish were obtained from Aquaculture Bureau of Sichuan Province and Aquaculture Bureau of Qinghai Province. The seven species, which are all the hypoxia-tolerant fish, live at different altitudes. The schizothoracine fish cover five genera and three groups. Tissue samples (heart and liver) were obtained and immediately infused with Trizol (TaKaRa) and conserved at −70°C until RNA extraction.

### RNA isolation, cDNA synthesis, and sequence amplification

RNA extraction was performed using the Trizol reagent according to the manufacturer’s instructions. The quality of RNA was determined on 1.2% EtBr-agarose gels, and the quantity (A_260_/A_280_) was measured with a UV spectrophotometer (Thermo Scientific Nanodrop 2000).

First, 1 μg of total RNA was used to synthesize first-strand cDNA using reverse transcriptase (Promega) in the presence of RNAse inhibitor (Invitrogen) with a cocktail of oligoT and dNTP (TaKaRa). The 5′-RACE-ready cDNA and 3′-RACE-ready cDNA were synthesized using a SMART™ RACE cDNA Amplification Kit (ClonTech) and FirstChoice® RLM-RACE Kit (Ambion), respectively. To obtain the intact cDNA, different tissue samples with the best quality RNA were chosen: heart for *Schizothorax prenanti*, *Gymnodiptychus pachycheilus*, and *Gymnocypris eckloni*, and liver for *Gymnocypris namensis*, *Schizopygopsis pylzovi*, *Platypharodon extremus*, and *Danio rerio*.

Universal primers (Additional file 6: Table S2) for amplifying partial cDNA fragments were designed using the Primer Premier 5 program against the conserved consensus sequences derived from multiple alignment of *hif-1αΑ/B* and *hif-2αΑ/B* sequences from *Schizothorax prenanti* (Prenant’s schizothoracin), *Danio rerio* (zebrafish), *Hypophthalmichthys molitrix* (silver carp), and *Hypophthalmichthys nobilis* (bighead carp), which were collected from Ensembl or National Center for Biotechnology Information (NCBI, Additional file 7: Table S3) [70]. First-strand cDNAs were used in the primary PCR amplification with the universal primers. The conditions for PCR amplification were an initial denaturation at 94°C for 3 min, 25 cycles of denaturation at 94°C for 30 s, annealing at 60°C for 30 s, and extension at 72°C for 2 min, and a final extension at 72°C for 10 min.

The partial sequences obtained with the universal primers were used to design the gene specific primers for 5′- and 3′-RACE reactions (Additional file 8: Table S4). RACE reactions were performed in accordance with the instructions in a SMART™ RACE cDNA Amplification Kit (ClonTech) and FirstChoice® RLM-RACE Kit (Ambion).

All PCR products were detected in 0.8% EtBr-agarose gels, and bands corresponding to *hif* fragments were excised and extracted with a DNA Gel Extraction Kit (Tiangen). The sequences were recombined with pMD18-T vector (TaKaRa), sequenced using ABI3730XL sequencer (Beijing Tianyi Huiyuan Bioscience and Technology Inc.), and assembled using SeqMan in DNASTAR Lasergene v7.1 software (DNASTAR Inc.). The assembled sequences were verified using the NCBI database. The full-length sequences of *hif-1αΑ/B* and *hif-2αΑ/B* were amplified using newly designed gene-specific primers to the 5′ and 3′ ends with the restriction enzyme sites (Additional file 9: Table S5).

### Sequence alignment and phylogenetic analysis

The alignment of the *hif-1α* and *hif-2α* coding sequences (corresponding to a total of 69 sequences and 3,090 bases pairs obtained after alignment) was based on notional translations of nucleotide sequences using ClustalX [71]. Additionally, the GTR + I (0.0821) + G (1.0813) model was adopted for the phylogeny by running PAUP4.0b10 and modeltest3.7 [72,73]. Finally, Mrbayes-3.1.2 with 2,000,000 iterations and RAxML with 1,000 nonparametric bootstrap replicates were used to construct a phylogenetic tree [42,43]. The sequences of sea squirt and amphioxus *hif-α* were used as outgroups.

### Model testing of selection pressures

To determine whether *hif* gene paralogs have undergone statistically significant differences in selection pressures, standard *branch-site* models in codeml software of PAML (version 4.6) and stochastic *branch-site* models in fitmodel (version 20140407) were used [48,49,74–78]. The two methods all employed ML estimates of the ratio of nonsynonymous to synonymous substitutions (*d*_N_/*d*_S_ = ω) and nested likelihood ratio tests (LRTs) on a phylogeny tree (Additional file 4: Figure S3). In standard *branch-site* models, only the Cyprinidae species which had all the four paralogs were included, and the clades corresponding to schizothoracine fish were marked as *foreground* branches.

### Recombined plasmid construction

The correct and intact *hif-1α* and *hif-2α* genes of *Danio rerio*, *Schizothorax prenanti*, *Gymnodiptychus pachycheilus*, and *Gymnocypris eckloni* were isolated from one of the TA clones and subcloned into pCMV-Myc vector (CloneTech) using special restriction enzyme sites (Additional file 6: Table S2). All recombined plasmids were verified by DNA sequencing.

### Cell culture

Human embryonic kidney (HEK) 293 T cells were cultured in Dulbecco’s modified Eagle’s medium (DMEM, Hyclone) supplemented with 10% fetal bovine serum (FBS, Hyclone) in a 5% CO_2_ incubator (Thermo). Hypoxia treatment was conducted in a hypoxia carbon dioxide incubator filled with 1% O_2_.

### Western blot analysis

HEK 293 T cells were seeded in 6-well plates for 24 h before transfection, and then transfected with 1.6 μg every plasmid (included empty vector and indicated Myc-tagged HIF-α constructs) using VigoFect (Vigorous). Six hours after transfection, the cells were divided into two groups and cultured under normoxic and hypoxic conditions (1% O_2_) for 18 h. Cells were lysed in RIPA buffer (Beyotime) and 1% PMSF was added (Beyotime). Then the lysate was mixed with 5× protein loading buffer (Beyotime), and boiled for 5 min. Protein samples were separated using the denaturing electrophoresis on 12% SDS-polyacrylamide vertical gels and transferred to a PVDF (polyvinylidene fluoride) membrane (Millipore). The membrane was blocked for 2 h in 5% nonfat dry milk in TBST (TBS: 7.3 g NaCl and 3.03 g Tris-Base diluted in 1,000 mL H_2_O, TBST: TBS supplemented with 0.1% Tween 20) at room temperature and incubated with primary antibody (c-Myc (9E10), Santa Cruz) that had been diluted 1:3,000 in 1% nonfat dry milk in TBST for 12 h at 4°C. The membrane was washed three times with TBST for 8 min each and incubated with the secondary antibody (HRP-labeled Goat Anti-Mouse IgG (H + L), Beyotime) that had been diluted 1:2,000 in 1% nonfat milk in TBST. The membrane was then washed, and the HIF-α protein was detected using enhanced chemiluminescence (ECL, Millipore) in an ImageQuant™ LAS 4000 mini (GE Healthcare). Tubulin was used as the internal control.

### Luciferase detection

HEK 293 T cells were seeded in 24-well plates for 24 h before transfection and then co-transfected with empty vector (control) or the indicated Myc-tagged HIF-α constructs, the HRE-luciferase reporter (provided by Professor Wuhan Xiao), and the pTK-*Renilla* luciferase reporter (used as an internal control) using VigoFect (Vigorous). Six hours after transfection, the cells were separated into two groups and cultured under normoxic and hypoxic conditions (1% O_2_) for 18 h. Luciferase activity was measured using a Dual-Luciferase Reporter Assay System (Promega).

## Additional files

Additional file 1: Table S1. Lengths of *hif* sequences. Additional file 2: Figure S1. Multiple sequence alignment of the deduced HIF-1α protein sequences. Dashes indicate the gaps inserted to facilitate alignment. All of the domains are indicated either inside a solid box or overlining. The two conserved proline residues within the ODD domain are indicated by the closed arrows, and the two conserved proline hydroxylation motif LxxLAP areas are indicated by dotted boxes. The open arrow indicates the asparagine residue (Asn-803 in human *hif-1α*) in C-TAD which controls HIF-1 binding to CBP/p300. Additional file 3: Figure S2. Phylogenetic tree constructed for chordate *hif-1α* and *hif-2α* gene. The phylogenetic tree constructed by MrBayes (left, 2,000,000 iterations) and RAxML (right, 1,000 nonparametric bootstrap replicates) with GTR + I (0.0821) + G (1.0813) model. Bayesian posterior probability values and maximum likelihood bootstrap values are indicated beside the branches. Additional file 4: Figure S3. Phylogenetic tree used to assess selection pressure. A) Phylogeny for standard *branch-site* models was constructed using concatenated *hif-1α* and *hif-2α* sequences by MrBayes with TVM + G (0.4427) model (2,000,000 iterations). B) The phylogeny for stochastic *branch-site* models was constructed using *hif-1αA* and *hif-1αB* sequences by MrBayes with GTR + G (0.7121) model (2,000,000 iterations). Additional file 5: Figure S4. Expression of HIF-α in HEK 293 T cells under hypoxic conditions. Generally speaking, there was more HIF-1α expression than that of HIF-2α expression after hypoxic treatment. Additional file 6: Table S2. Universal primers used in this study. Additional file 7: Table S3. Database ID of the sequences used in this study. Additional file 8: Table S4. Gene-specific primers for 5′-RACE and 3′-RACE. Additional file 9: Table S5. Gene-specific primers with restriction enzyme sites for full-length sequences.

## Abbreviations

HIF: Hypoxia-inducible factor
HIE: Hypoxia-inducible enhancer
HRE: Hypoxia-response element
bHLH-PAS: Basic helix-loop-helix-per-ARNT-sim
ODDD: Oxygen-dependent degradation domains
TAD: Transactivation activation domain
RACE: Rapid amplification of cDNA ends
PHD: Prolyl-4-hydroxylase
VHL: Von-Hippel-Lindau tumor suppressor
NLS: Nuclear localization signal
BEB: Bayes empirical Bayes
LRTs: Likelihood ratio tests
HEK: Human embryonic kidney
DMEM: Dulbecco’s modified Eagle’s medium
FBS: Fetal bovine serum
SDS: Sodium dodecyl sulfate
PVDF: Polyvinylidene fluoride
ECL: Enhanced chemiluminescence
