# Supplementary material for: Analysis of hypoxia-inducible factor alpha polyploidization reveals adaptation to Tibetan plateau in the evolution of schizothoracine fish
Source: BMC Evol Biol. 2014 Aug 28;14:192. doi: 10.1186/s12862-014-0192-1 (PMC4162920; doi:10.1186/s12862-014-0192-1)
Supplement: Additional file 1: Table S1. — Lengths of hif sequences. [file 12862_2014_192_MOESM1_ESM.docx]

**Additional file 1** – **Table** **S1 Lengths of *hif* sequences**

| Group | Genus name | *Latin name* | Lengths of gene (bp) | | | | Lengths of coded amino acid | | | |
| --- | --- | --- | --- | --- | --- | --- | --- | --- | --- | --- |
|  |  |  | *hif1αA* | *hif1αB* | *hif2αA* | *hif2αB* | *hif1αA* | *hif1αB* | *hif2αA* | *hif2αB* |
| Ⅰ | Schizothorax | *Schizothorax prenanti* | 2136 | 2313 | 2353 | 2502 | 711 | 770 | 844 | 833 |
| Ⅱ | Gymnodiptychus | *Gymnodiptychus pachycheilus* | 2154 | 2316 | 2535 | 2481 | 717 | 771 | 844 | 821 |
| Ⅲ | Gymnocypris | *Gymnocypris namensis* | 2175 | 2322 | 2523 | 2493 | 724 | 773 | 840 | 830 |
|  |  | *Gymnocypris eckloni eckloni* | 2175 | 2322 | 2523 | 2493 | 724 | 773 | 840 | 830 |
|  | Schizopygopsis | *Schizopygopsis pylzovi* | 2175 | 2322 | 2523 | 2493 | 724 | 773 | 840 | 830 |
|  | Platypharodon | *Platypharodon extremus* | 2175 | 2322 | 2523 | 2493 | 724 | 773 | 840 | 830 |
