# Supplementary figures and images for: Analysis of hypoxia-inducible factor alpha polyploidization reveals adaptation to Tibetan plateau in the evolution of schizothoracine fish
Source: BMC Evol Biol. 2014 Aug 28;14:192. doi: 10.1186/s12862-014-0192-1 (PMC4162920; doi:10.1186/s12862-014-0192-1)

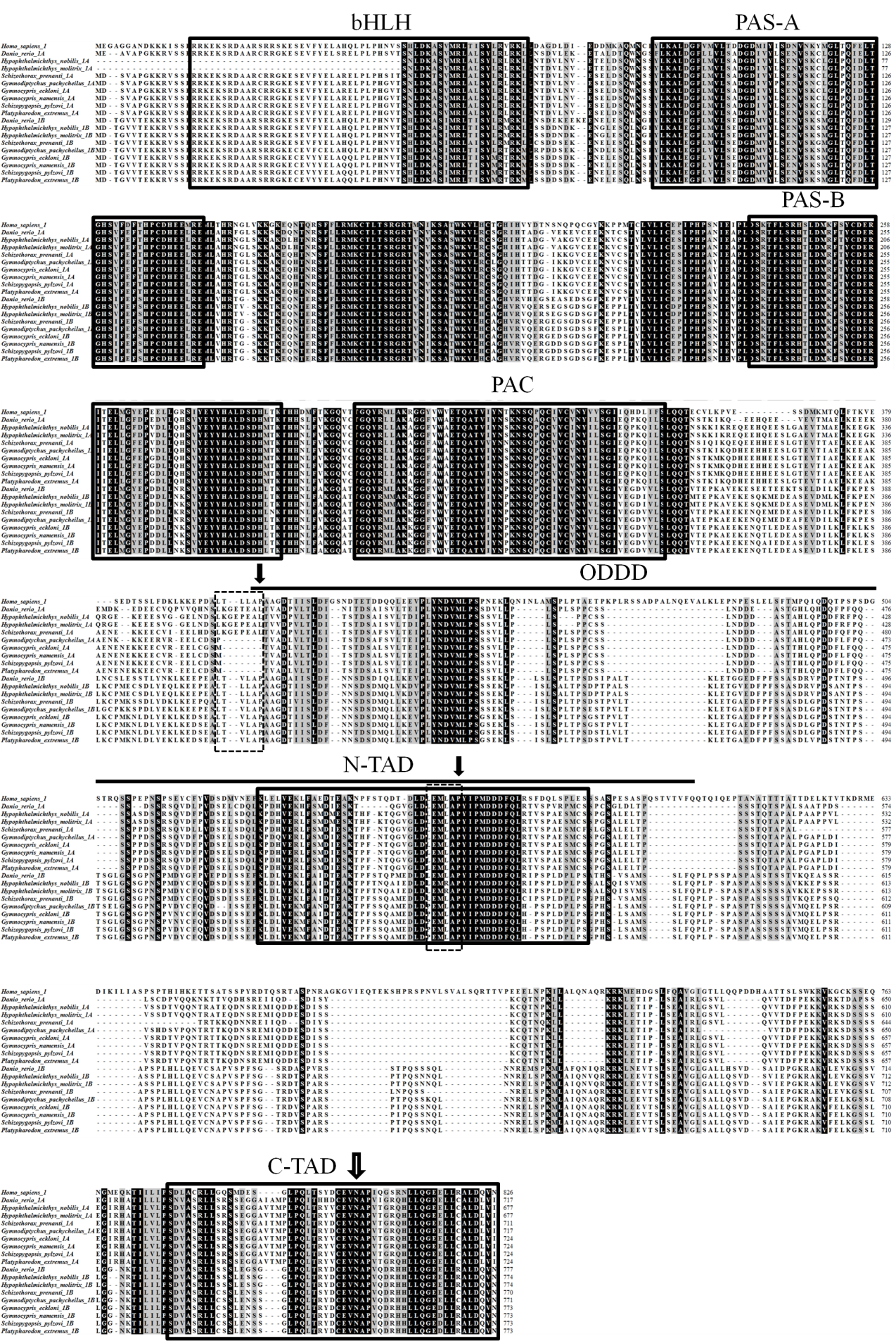


**Additional file 2 – Fig.** **S1 Multiple sequence alignment of the deduced HIF-1α protein sequences.**

Supplement: Additional file 2: Figure S1. — Multiple sequence alignment of the deduced HIF-1α protein sequences. Dashes indicate the gaps inserted to facilitate alignment. All of the domains are indicated either inside a solid box or overlining. The two conserved proline residues within the ODD domain are indicated by the closed arrows, and the two conserved proline hydroxylation motif LxxLAP areas are indicated by dotted boxes. The open arrow indicates the asparagine residue (Asn-803 in human hif-1α) in C-TAD which controls HIF-1 binding to CBP/p300. [file 12862_2014_192_MOESM2_ESM.docx]
