# Supplementary material for: Analysis of hypoxia-inducible factor alpha polyploidization reveals adaptation to Tibetan plateau in the evolution of schizothoracine fish
Source: BMC Evol Biol. 2014 Aug 28;14:192. doi: 10.1186/s12862-014-0192-1 (PMC4162920; doi:10.1186/s12862-014-0192-1)
Supplement: Additional file 3: Figure S2. — Phylogenetic tree constructed for chordate hif-1α and hif-2α gene. The phylogenetic tree constructed by MrBayes (left, 2,000,000 iterations) and RAxML (right, 1,000 nonparametric bootstrap replicates) with GTR + I (0.0821) + G (1.0813) model. Bayesian posterior probability values and maximum likelihood bootstrap values are indicated beside the branches. [file 12862_2014_192_MOESM3_ESM.docx]

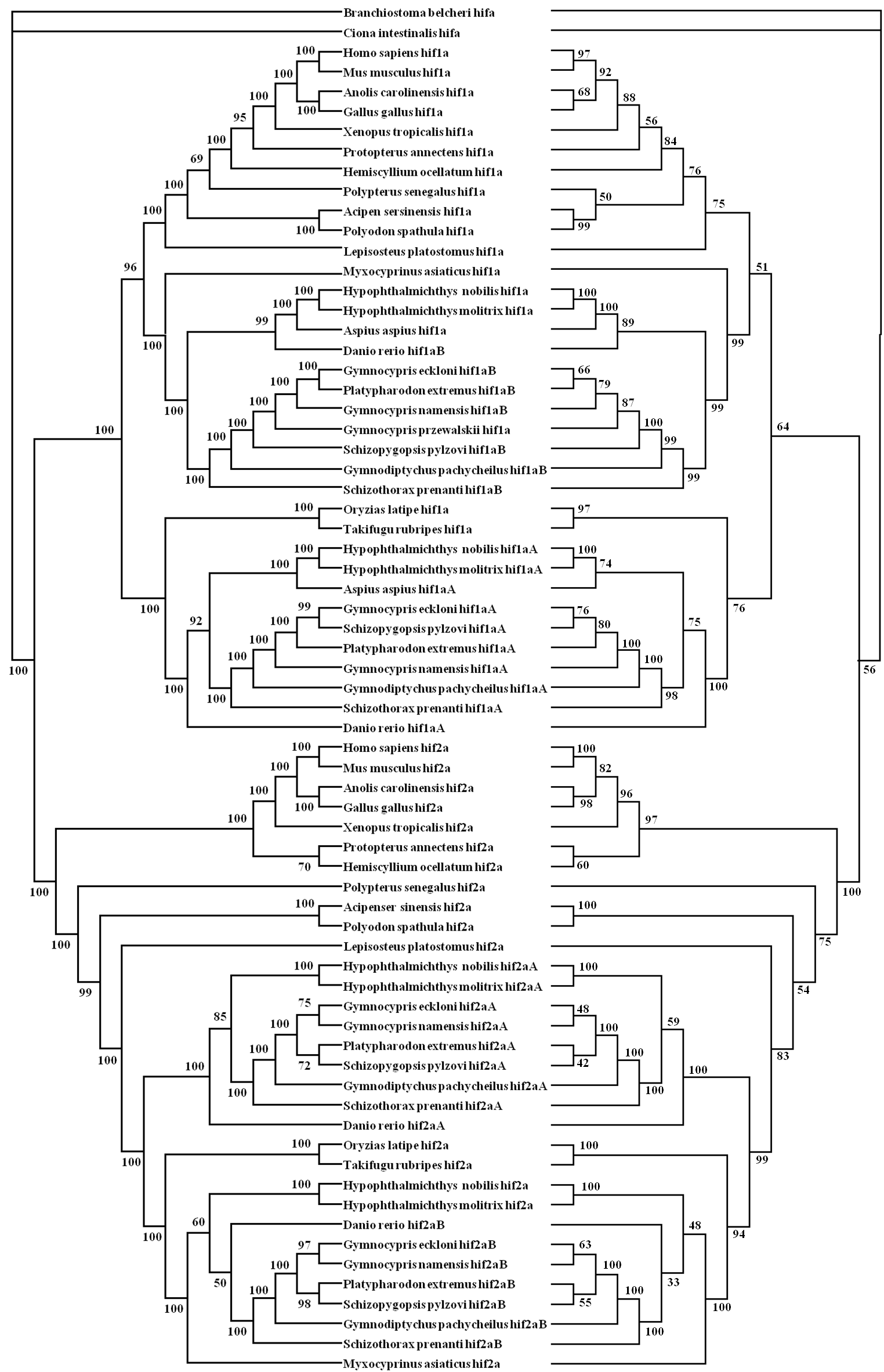


**Additional file 3** – **Fig.** **S2 Phylogenetic tree constructed for chordate *hif-1α* and *hif-2α* gene.**

The phylogenetic tree was constructed by MrBayes (left, 2,000,000 iterations) and RAxML (right, 1,000 nonparametric bootstrap replicates) with GTR + I + G model. Bayesian posterior probability values and maximum likelihood bootstrap values are indicated beside the branches.
