# Supplementary material for: Analysis of hypoxia-inducible factor alpha polyploidization reveals adaptation to Tibetan plateau in the evolution of schizothoracine fish
Source: BMC Evol Biol. 2014 Aug 28;14:192. doi: 10.1186/s12862-014-0192-1 (PMC4162920; doi:10.1186/s12862-014-0192-1)
Supplement: Additional file 4: Figure S3. — Phylogenetic tree used to assess selection pressure. A) Phylogeny for standard branch-site models was constructed using concatenated hif-1α and hif-2α sequences by MrBayes with TVM + G (0.4427) model (2,000,000 iterations). B) The phylogeny for stochastic branch-site models was constructed using hif-1αA and hif-1αB sequences by MrBayes with GTR + G (0.7121) model (2,000,000 iterations). [file 12862_2014_192_MOESM4_ESM.docx]

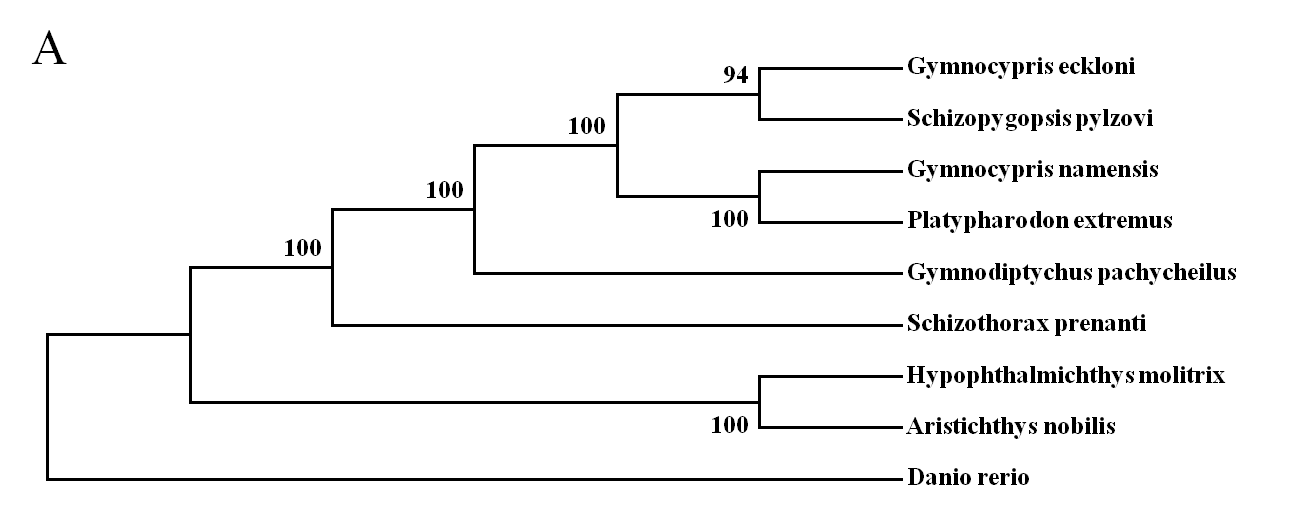


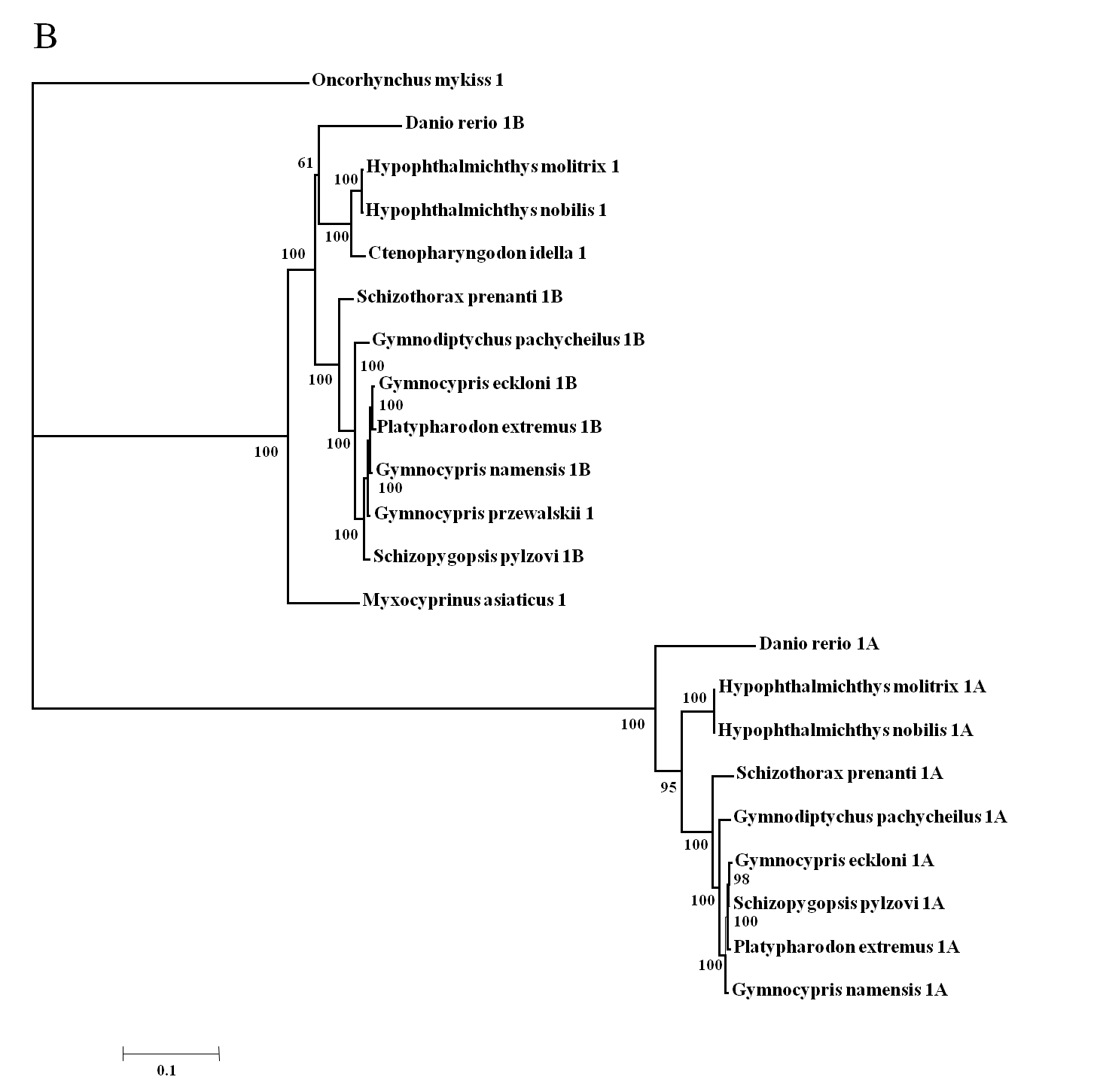


**Additional file 4** – **Fig.** **S3 Phylogenetic tree used to assess selection pressure.**

A) Phylogeny for standard *branch-site* models was constructed using concatenated *hif-1α* and *hif-2α* sequences by MrBayes with TVM + G (0.4427) model (2,000,000 iterations). B) The phylogeny for stochastic *branch-site* models was constructed using hif-1αA and hif-1αB sequences by Mrbayes with GTR + G (0.7121) model (2,000,000 iterations).
