# Supplementary material for: Analysis of hypoxia-inducible factor alpha polyploidization reveals adaptation to Tibetan plateau in the evolution of schizothoracine fish
Source: BMC Evol Biol. 2014 Aug 28;14:192. doi: 10.1186/s12862-014-0192-1 (PMC4162920; doi:10.1186/s12862-014-0192-1)
Supplement: Additional file 5: Figure S4. — Expression of HIF-α in HEK 293 T cells under hypoxic conditions. Generally speaking, there was more HIF-1α expression than that of HIF-2α expression after hypoxic treatment. [file 12862_2014_192_MOESM5_ESM.docx]

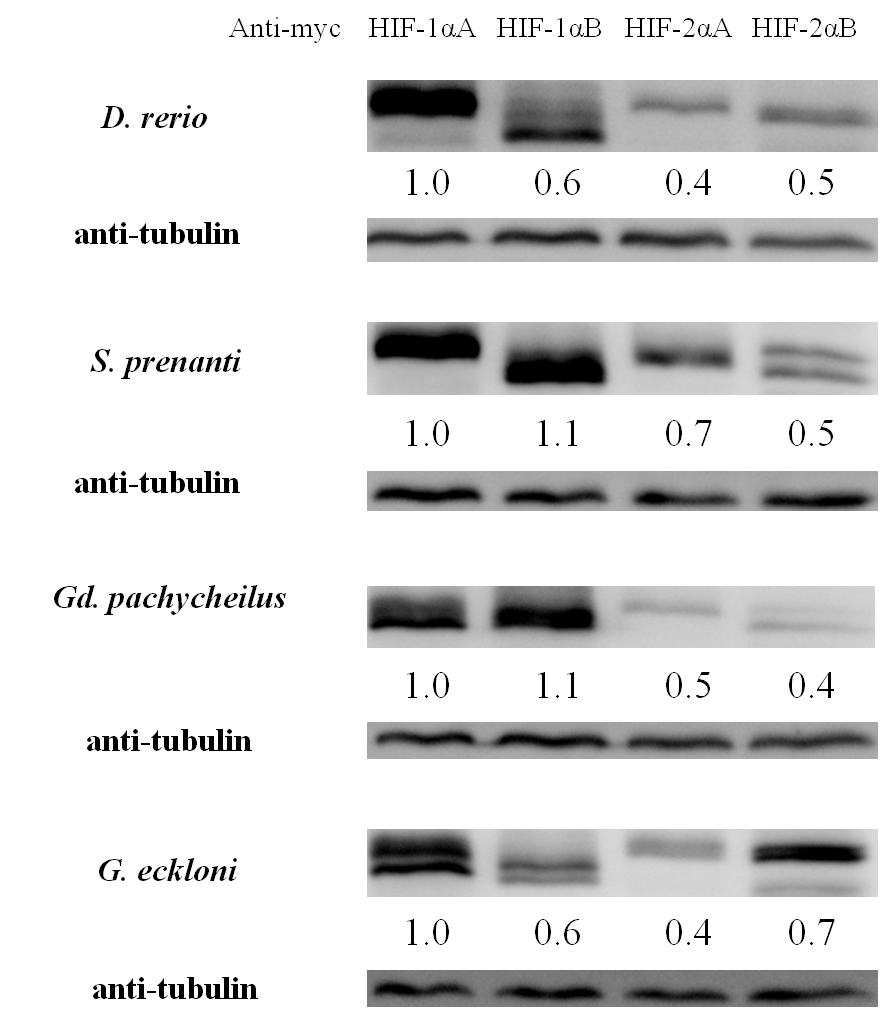


**Additional file 5** – **Fig.** **S4 Expression of HIF-α in HEK 293T cells under hypoxic conditions.**

Generally speaking, there was more HIF-1α expression than that of HIF-2α after hypoxic treatment.
