# Supplementary material for: Analysis of hypoxia-inducible factor alpha polyploidization reveals adaptation to Tibetan plateau in the evolution of schizothoracine fish
Source: BMC Evol Biol. 2014 Aug 28;14:192. doi: 10.1186/s12862-014-0192-1 (PMC4162920; doi:10.1186/s12862-014-0192-1)
Supplement: Additional file 6: Table S2. — Universal primers used in this study. [file 12862_2014_192_MOESM6_ESM.docx]

**Additional file 6** – **Table** **S2 Universal primers used in this study**

| Primer name | Sequence |
| --- | --- |
| hif1A-f | CTCTAACCTGGATAAAGCATC |
| hif1A-r | CTCTGGAAAATCGGTAACAACC |
| hif1A-r2 | TTCTGATGATGAAGCATCTGA |
| hif1B-f | CCTGTCTGAAGATGGAGACAT |
| hif1B-r | TGCACGGGAGCGTTGACTT |
| hif2A-f | CAACTCAGGCCGCACAGT |
| hif2A-r | TGACTTATCATGCCTGTGT |
| hif2A-r2 | GTTGACTTATCATGCCTGAGT |
| hif2B-f | AAGTCACTGGGCGGCTTCAT |
| hif2B-r | GCGTCAGTTCAGGCAGAGAGT |
