# Supplementary material for: Analysis of hypoxia-inducible factor alpha polyploidization reveals adaptation to Tibetan plateau in the evolution of schizothoracine fish
Source: BMC Evol Biol. 2014 Aug 28;14:192. doi: 10.1186/s12862-014-0192-1 (PMC4162920; doi:10.1186/s12862-014-0192-1)
Supplement: Additional file 7: Table S3. — Database ID of the sequences used in this study. [file 12862_2014_192_MOESM7_ESM.docx]

**Additional file 7** – **Table** **S3 Database ID of the sequences used in this study**

| Species | Gene | Accession number | New sequences accession number |
| --- | --- | --- | --- |
| *Acipenser gueldenstaedtii* | hif1α | GenBank: EF100701.1 |  |
| *Acipenser sinensis* | hif1α | GenBank: JQ031037.1 |  |
|  | hif2α | GenBank: JQ031045.1 |  |
| *Anolis carolinensis* | hif1α | Ensembl: ENSACAT00000016241 |  |
|  | hif2α | Ensembl: ENSACAT00000004116 |  |
| *Asipius aspius* | hif1α | GenBank: EF100702.1 |  |
|  | hif1αA | GenBank: JQ027712.1 |  |
| *Branchiostoma belcheri* | hifα | GenBank: HM188448 |  |
| *Carassius carassius* | hif1α | GenBank: DQ306727.1 |  |
| *Ciona intestinalis* | hifα | GenBank: NM_001078263.1 |  |
| *Ctenopharyngodon idella* | hif1α | GenBank: AY450269.2 |  |
| *Danio rerio* | hif1αA | Ensembl: ENSDART00000044282 | GenBank: KJ679875 |
|  | hif1αB | Ensembl: ENSDART00000018500 |  |
|  | hif2αA | Ensembl: ENSDART00000024415 |  |
|  | hif2αB | Ensembl: ENSDART00000080382 |  |
| *Gallus gallus* | hif1α | GenBank: NM_204297.1 |  |
|  | hif2α | GenBank: NM_204807.1 |  |
| *Gymnocypris eckloni* | hif1αA |  | GenBank: KJ679878 |
|  | hif1αB |  | GenBank: KJ679884 |
|  | hif2αA |  | GenBank: KJ679890 |
|  | hif2αB |  | GenBank: KJ679896 |
| *Gymnocypris namensis* | hif1αA |  | GenBank: KJ679879 |
|  | hif1αB | GenBank: JQ031039.1 | GenBank: KJ679885 |
|  | hif2αA |  | GenBank: KJ679891 |
|  | hif2αB | GenBank: JQ031047.1 | GenBank: KJ679897 |
| *Gymnodiptychus pachycheilus* | hif1αA |  | GenBank: KJ679877 |
|  | hif1αB |  | GenBank: KJ679883 |
|  | hif2αA |  | GenBank: KJ679889 |
|  | hif2αB |  | GenBank: KJ679895 |
| *Gymnodiptychus przewalskii* | hif1α | GenBank: AY745735.1 |  |
| *Hemiscyllium ocellatum* | hif1α | GenBank: EU262661.1 |  |
|  | hif2α | GenBank: GQ152300.1 |  |
| *Homo sapiens* | hif1α | Ensembl: ENST00000337138 |  |
|  | hif2α | Ensembl: ENST00000263734 |  |
| *Hypophthalmichthys molitrix* | hif1αA |  | GenBank: KJ697677 |
|  | hif1αB | GenBank: HM146310.1 |  |
|  | hif2αA |  | GenBank: KJ697679 |
|  | hif2αB | GenBank: JQ031049.1 |  |
| *Hypophthalmichthys nobilis* | hif1αA |  | GenBank: KJ697678 |
|  | hif1αB | GenBank: JQ031036.1 |  |
|  | hif2αA |  | GenBank: KJ697680 |
|  | hif2αB | GenBank: JQ031044.1 |  |
| *Lepisosteus platostomus* | hif1α | GenBank: JQ031038.1 |  |
|  | hif2α | GenBank: JQ031046.1 |  |
| *Mus musculus* | hif1α | Ensembl: ENSMUST00000021530 |  |
|  | hif2α | Ensembl: ENSMUST00000024954 |  |
| *Megalobrama amblycephala* | hif1α | GenBank: GU363498.1 |  |
| *Myxocyprinus asiaticus* | hif1α | GenBank: HQ432957.1 |  |
|  | hif2α | GenBank: HQ432955.1 |  |
| *Oncorhynchus mykiss* | hif1α | GenBank: AF304864.1 |  |
| *Oryzias latipe* | hif1α | GenBank: XM_004082546.1 |  |
|  | hif2α | GenBank: XM_004077268.1 |  |
| *Platypharodon extremus* | hif1αA |  | GenBank: KJ679881 |
|  | hif1αB |  | GenBank: KJ679887 |
|  | hif2αA |  | GenBank: KJ679893 |
|  | hif2αB |  | GenBank: KJ679899 |
| *Polyodon spathula* | hif1α | GenBank: JQ031041.1 |  |
|  | hif2α | GenBank: JQ031048.1 |  |
| *Polypterus senegalus* | hif1α | GenBank: JQ031035.1 |  |
|  | hif2α | GenBank: JQ031043.1 |  |
| *Protopterus annectens* | hif1α | GenBank: JQ031040.1 |  |
|  | hif2α | GenBank: JQ031058.1 |  |
| *Schizopygopsis pylzovi* | hif1αA |  | GenBank: KJ679880 |
|  | hif1αB |  | GenBank: KJ679886 |
|  | hif2αA |  | GenBank: KJ679892 |
|  | hif2αB |  | GenBank: KJ679898 |
| *Schizothorax prenanti* | hif1αA |  | GenBank: KJ679876 |
|  | hif1αB | GenBank: JQ031042.1 | GenBank: KJ679882 |
|  | hif2αA |  | GenBank: KJ679888 |
|  | hif2αB | GenBank: JQ031050.2 | GenBank: KJ679894 |
| *Takifugu rubripes* | hif1α | Ensembl: ENSTRUT00000030720 |  |
|  | hif2α | Ensembl: ENSTRUT00000013655 |  |
| *Xenopus_tropicalis* | hif1α | Ensembl: ENSXETT00000031610 |  |
|  | hif2α | GenBank: BC074648.1 |  |
