# Supplementary material for: Analysis of hypoxia-inducible factor alpha polyploidization reveals adaptation to Tibetan plateau in the evolution of schizothoracine fish
Source: BMC Evol Biol. 2014 Aug 28;14:192. doi: 10.1186/s12862-014-0192-1 (PMC4162920; doi:10.1186/s12862-014-0192-1)
Supplement: Additional file 8: Table S4. — Gene-specific primers for 5′-RACE and 3′-RACE. [file 12862_2014_192_MOESM8_ESM.docx]

**Additional file 8** – **Table** **S4 Gene-specific primers for 5**'-**RACE and 3'**-**RACE**

| Primer name | Sequence |
| --- | --- |
| hif1A-5R-IP | CAATAGAAAACTCCTGTTTGTGTG |
| hif1A-5R-IP2 | TCAGCAGGAAACTTCTGTTTGTGT |
| hif1A-5R-OP | ACCAGGTACGTTGAACACACATT |
| hif1B-5R-IP | TCCTGTCCTGTGGACCAGCAT |
| hif1B-5R-OP | ACAGATGAGCACAAGGTTAGA |
| hif2A-5R-IP | ACTGGGTTTGCAGGACTGTGCG |
| hif2A-5R-OP | TGCCATACAAAGTTCCTGATTTGAC |
| hif2B-5R-IP | ATGCCGGCTTTGAGGCTGAGGT |
| hif2B-5R-OP | ACAGCCGTTATACACCTTCAGGT |
| hif1A-3R-IP | CTCCAGCAGCACACAAACTGC |
| hif1A-3R-OP | TGTGGATTCAGAATTAAGTGACCA |
| hif1B-3R-IP | TGTGGACAGTGCTATAGAGCCT |
| hif1B-3R-OP | CTGCACCTGCTGCAGGAGGT |
| hif2A-3R-IP | CAAAGGAAGCTCAGAGGATCT |
| hif2A-3R-OP | CAGAAACTCATGAGTGTGGGTCA |
| hif2B-3R-IP | GAATCAGGCCGGACAGGCGA |
| hif2B-3R-OP | CATGGAGAACTTTGTGCAGAACT |
