# Supplementary material for: Analysis of hypoxia-inducible factor alpha polyploidization reveals adaptation to Tibetan plateau in the evolution of schizothoracine fish
Source: BMC Evol Biol. 2014 Aug 28;14:192. doi: 10.1186/s12862-014-0192-1 (PMC4162920; doi:10.1186/s12862-014-0192-1)
Supplement: Additional file 9: Table S5. — Gene-specific primers with restriction enzyme sites for full-length sequences. [file 12862_2014_192_MOESM9_ESM.docx]

**Additional file 9** – **Table** **S5 Gene-specific primers with restriction enzyme sites for full-length sequences**

| Primer name | Sequence | Restriction enzyme sites |
| --- | --- | --- |
| hif1A-f (Spr/Gp/Ge) | GCTAGTCGACCATGGATTCAGTCGCACCTGGGA | SalⅠ |
| hif1A-f (Dr) | GCTAGTCGACCATGGAGGCAGTCGCACCTGG | SalⅠ |
| hif1A-r (Spr/Gp/Ge) | TAGCAGATCTCTTAAATAACTAGGTCCAAGGCACACAGTAG | Bgl Ⅱ |
| hif1A-r (Dr) | TAGCAGATCTCTTAAATAACTAAATCCAAGGCACAAAGAAGC | Bgl Ⅱ |
| hif1B-f (Spr/Gp/Ge) | GCTAGTCGACCATGGATACTGGAGTTGTCACTGAAAAGAAAAG | SalⅠ |
| hif1B-f (Dr) | GCTAGTCGACCATGGATACTGGAGTTGTCACTGAAAAGA | SalⅠ |
| hif1B-r (Spr/Gp) | TAGCAGATCTCTCAGTTGACTTGGTCCAGAGCACACA | Bgl Ⅱ |
| hif1B-r (Ge) | TAGCAGATCTCTCAGTTGACTTGGTCCAGAGCACG | Bgl Ⅱ |
| hif1B-r (Dr) | TAGCAGATCTCTCAGTTGACTTGGTCCAGAGCACG | Bgl Ⅱ |
| hif2A-f (Spr/Ge) | ATGCGAATTCGGATGACAGCCGAGAAAGAGAAAAAAAG | EcoR Ⅰ |
| hif2A-f (Gp) | ATGCGAATTCGGATGACAGCCGAGAGAGAGAGAAAAAG | EcoR Ⅰ |
| hif2A-f (Dr) | ATGCGAATTCGGATGACAGCCGAGAGAGAGAAAAAGAGG | EcoR Ⅰ |
| hif2A-r (Spr) | CGATAGATCTCCTATGTGGACTGGTCCAAAGCTCTC | Bgl Ⅱ |
| hif2A-r (Gp) | CGATCTCGAGACTATGTGGTCTGGTCCAAAGCTCTTAG | Xho Ⅰ |
| hif2A-r (Ge) | CGATAGATCTCCTATGTGGTCTGGTCCAAAGCTCTC | Bgl Ⅱ |
| hif2A-r (Dr) | CGATAGATCTCTTATGTGGACTGGTCTAGGGCTCTCA | Bgl Ⅱ |
| hif2B-f (Spr) | ATGCGAATTCGAATGACAGCGGAGAAAGAGAAGAAGAGGT | EcoR Ⅰ |
| hif2B-f (Gp) | ATGCGAATTCGAATGACAGTGGAGAAGGAGAAGAGGTGC | EcoR Ⅰ |
| hif2B-f (Ge) | ATGCGAATTCGAATGACAGCGGAGAAGGAGAAGAAGAGG | EcoR Ⅰ |
| hif2B-f (Dr) | ATGCGAATTCGAATGACAGCTGAGAAAGAGAAGAAGAGGTG | EcoR Ⅰ |
| hif2B-r (Spr/Gp) | CGATGTCGACCGCTAAGTAGCCTGGTCTAATGCTCTTAGGAGGT | SalⅠ |
| hif2B-r (Ge) | CGATGTCGACCGCTAAGTGGCCTGGTCTAATGCTCTTAGGA | SalⅠ |
| hif2B-r (Dr) | CGATGTCGACCGCTAAGTCGCCTGGTCTAATGCTCTTAGG | SalⅠ |
